# CitE Enzymes Are Essential for *Mycobacterium tuberculosis* to Establish Infection in Macrophages and Guinea Pigs

**DOI:** 10.3389/fcimb.2018.00385

**Published:** 2018-11-06

**Authors:** Garima Arora, Deepika Chaudhary, Saqib Kidwai, Deepak Sharma, Ramandeep Singh

**Affiliations:** ^1^Tuberculosis Research Laboratory, Vaccine and Infectious Disease Research Centre, Translational Health Science and Technology Institute, Faridabad, India; ^2^Symbiosis School of Biological Sciences, Symbiosis International University, Lavale, India; ^3^Manipal Academy of Higher Education, Manipal, India; ^4^Department of Biotechnology, Indian Institute of Technology Roorkee, Roorkee, India

**Keywords:** *Mycobacterium tuberculosis*, reverse TCA, β-subunit of citrate lyase, virulence, oxidative stress

## Abstract

Bacterial citrate lyase activity has been demonstrated in various eukaryotes, bacteria and archaea, underscoring their importance in energy metabolism of the cell. While the bacterial citrate lyase comprises of three different subunits, *M. tuberculosis* genome lacks CitD and CitF subunits of citrate lyase complex but encodes for 2 homologs of CitE subunits, Rv2498c and Rv3075c. Using temperature sensitive mycobacteriophages, we were able to generate both single and double *citE* mutant strains of *M. tuberculosis*. The survival experiments revealed increased susceptibility of the double mutant strain to oxidative stress in comparison to the parental strain. Also, simultaneous deletion of both *citE1* and *citE2* in *M. tuberculosis* genome resulted in impairment of intracellular replication in macrophages. The double mutant strain displayed reduced growth in lungs and spleens of guinea pigs. This is the first study demonstrating that *M. tuberculosis* critically requires CitE subunits of citrate lyase for pathogenesis. Taken together, these findings position these enzymes as potential targets for development of anti-tubercular small molecules.

## Introduction

Tuberculosis (TB) is a leading cause of morbidity and mortality with an estimated 1.7 billion people infected with the pathogen worldwide (WHO, 2017). The association of TB with other factors, such as HIV, diabetes, smoking, alcoholism, and malnutrition further complicates TB treatment and control. The first-line TB drugs are becoming less useful due to issues of non-compliance and emergence of drug resistant strains. *Mycobacterium tuberculosis* (*M. tuberculosis*) has evolved its metabolic networks to adapt and survive inside the host macrophages (Gomez and McKinney, 2004; Rohde et al., 2007). Numerous studies have shown that *M. tuberculosis* utilizes both conventional and non-conventional carbon metabolic pathways to persist and establish infection in the host. The strains lacking enzymes involved in either central carbon metabolism or methyl citrate cycle or glyoxylate cycle are attenuated for growth in macrophages and mice tissues (McKinney et al., 2000; Muñoz-Elías and McKinney, 2005; Muñoz-Elías et al., 2006; Marrero et al., 2010; Puckett et al., 2014; Trujillo et al., 2014). Further, *M. tuberculosis* is also able to utilize fatty acids as principal carbon source during *in vivo* infection (Bloch and Segal, 1956; Pandey and Sassetti, 2008).

The tricarboxylic acid cycle (TCA) plays an essential role in cellular metabolism by providing reducing equivalents for energy generation and precursors for lipids and amino acids biosynthesis. It has been reported that a variant of TCA cycle is operational in *M. tuberculosis* as it lacks a conventional α-ketoglutarate dehydrogenase (Tian et al., 2005). Tian et al., reported that ketoglutarate decarboxylase catalyzes thiamine pyrophosphate dependent conversion of α-ketoglutarate to succinic semialdehyde which is subsequently oxidized to succinate by succinic semialdehyde dehydrogenase (Tian et al., 2005). It has also been demonstrated that in low oxygen conditions, transcripts of enzymes involved in reverse TCA cycle, such as fumarate reductase were upregulated and this induction was associated with accumulation of succinate in extracellular mileu (Watanabe et al., 2011). The reverse TCA cycle was first reported in green sulfur bacterium, *Chlorobium thiasulfatophilum* that utilizes this pathway for CO_2_ fixation photoautotrophically (Tang and Blankenship, 2010). Most of the enzymes are common in forward and reverse TCA cycle except for ATP citrate lyase, α-ketoglutarate:ferredoxin reductase and fumarate reductase. In eukaryotes, ATP dependent citrate lyase is a tetramer of identical subunits that converts citrate into acetyl-CoA and oxaloacetate. In prokaryotes, citrate lyase activity is ATP independent and required for anaerobic fermentation of citrate (Bott, 1997). Bacterial citrate lyase comprises six copies of each subunit, α, β, and γ. The γ-subunit (CitD) functions as an acyl carrier protein (ACP) and contains coenzyme A (CoA) derivative as a prosthetic group (Schneider et al., 2000). The α-subunit (CitF) functions as an acyl transferase and is responsible for the formation of citryl-ACP intermediate (Dimroth and Eggerer, 1975). CitE, the β-subunit cleaves citryl-CoA into oxaloacetate and acetyl-CoA (Dimroth and Eggerer, 1975). Enzymes belonging to the CitE superfamily are either part of citCDEF(X)G operon or are stand alone genes or part of clusters that are unrelated to citrate lyase operon (Bott and Dimroth, 1994; Schneider et al., 2000; Goulding et al., 2007). In *Cryptococcus neoformans*, strains lacking ATP citrate lyase showed defective growth, production of virulence factors, increased susceptibility to fluconazole and decreased survival in macrophages and mice model of infection (Griffiths et al., 2012). In *Pseudomonas fluorescens*, exposure to nitrosative stress invoked activation of citrate lyase, phosphoenolpyruvate kinase and pyruvate diphosphate kinase resulting in conversion of citrate into pyruvate and ATP (Auger et al., 2011; Auger and Appanna, 2015). RipC, a β-subunit of citrate lyase has been proposed as a putative CoA- or CoA-derivative binding protein and predicted to be important for virulence of *Yersinia pestis* (Pujol et al., 2005; Torres et al., 2012). CLYBL, citrate lyase beta-like protein, human mitochondrial enzyme, possesses malate/β-methyl malate synthase activity resulting in formation of malate or β-methyl malate from glyoxylate and acetyl-CoA or propionyl-CoA, respectively (Strittmatter et al., 2014).

The genome of *M. tuberculosis* lacks homologs for α and γ subunits of citrate lyase but encodes for two homologs of β-subunit of citrate lyase, Rv2498c (CitE1) and Rv3075c (CitE2) (Cole and Barrell, 1998; Cole et al., 1998). The three dimensional structure of CitE1 bound with oxaloacetate and magnesium has been reported but the exact role of this enzyme in *M. tuberculosis* physiology and virulence has not been explored so far (Goulding et al., 2007). Here, we sought to biochemically and functionally characterize CitE homologs from *M. tuberculosis*. Using purified proteins, we show that both CitE1 and CitE2 enzymes non-specifically degraded acetyl-CoA and propionyl-CoA. The transcript levels of both *citE1* and *citE2* were increased in *M. tuberculosis* upon exposure to low oxygen and nitrosative stress conditions. The double mutant strain exhibited increased susceptibility upon exposure to oxidative and detergent stress in comparison to the parental strain. The double mutant strain was also attenuated for growth in macrophages and guinea pigs. Taken together, we conclusively show that the CitE enzymes are important for *M. tuberculosis* pathogenesis and might be useful as drug targets.

## Results

### *M. tuberculosis* genome encodes for 2 homologs of cite subunits of citrate lyase enzyme

**Figure 1:**
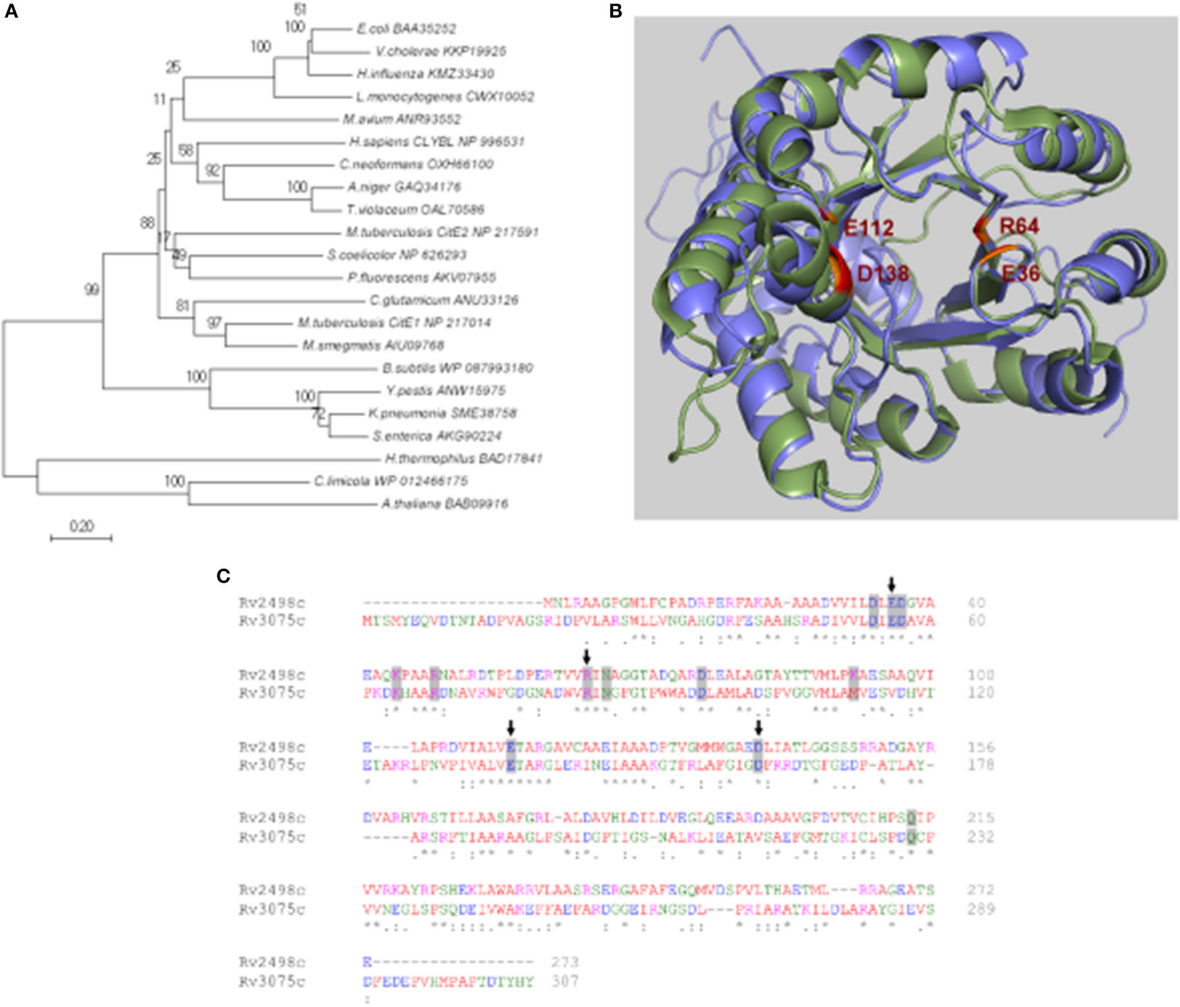
*In silico* analysis of citrate lyase proteins. **(A)** The phylogenetic tree was constructed using Minimum-Evolution method in MEGA7 software and distances are in the units of number of amino acid substitutions per site. The tree is drawn to scale, with branch lengths in the same units as those of the evolutionary distances used to infer the phylogenetic tree. The branches are labeled with the protein accession number along with organism name. The bootstrap consensus tree inferred from 1,000 replicates is taken to represent the evolutionary history of the taxa. **(B)** Alignment of the experimentally determined structure of CitE1 (green) and the modeled structure of CitE2 (blue). The catalytic site residues E36, R64, E112, and D138 of CitE1 have been highlighted in red and the corresponding residues of CitE2 have been shown in orange. **(C)** Sequence alignment of Rv2498c (CitE1) and Rv3075c (CitE2) by Clustal Omega. The catalytic site residues E36, R64, E112, and D138 of CitE1 have been marked by arrows. The residues conserved in various citrate lyases have been shaded in gray (Goulding et al., 2007).

The detailed bioinformatic analysis and homology searches revealed that the genome of *M. tuberculosis* lacks homologs for α- and γ-subunits of citrate lyase. However, the genome of *M. tuberculosis* encodes for 2 homologs of CitE (β-subunit) proteins, Rv2498c (CitE1) and Rv3075c (CitE2). Phylogenetic analysis revealed that *M. tuberculosis* CitE1 homolog was similar to homologs from *M. smegmatis* and *Corynebacterium glutamicum* while *M. tuberculosis* CitE2 homolog was similar to those from *Streptomyces coelicolor* and *Pseudomonas fluorescens* (Figure 1A). The crystal structure of *M. tuberculosis* CitE1 revealed that this protein is trimeric and possesses (β/α)_8_ TIM barrel fold similar to other CitE enzymes with an additional α-helix (Goulding et al., 2007). We next constructed homology model for CitE2 enzyme based on the available structures of CitE protein from *Yersinia pestis* (3QLL.pdb) and malyl-CoA lyase from *Methylobacterium extorquens* (4ROQ.pdb) using Modeller software (Sali and Blundell, 1993). These templates 3QLL and 4ROQ were chosen since their protein sequences showed maximum identity as well as query coverage with *M. tuberculosis* CitE2 enzyme (Supplementary Figure 1). The modeled structure of CitE2 almost completely aligned with the experimentally determined structure of CitE1 (1Z6K.pdb) and root mean square deviation between these two structures was 0.977 Å (Figure 1B). These two enzymes from *M. tuberculosis* share an identity of 33% among themselves (Figure 1C). Notably, the catalytic site formed by the residues was similar in CitE1 (E36, R64, E112, and D138) and CitE2 (E56, R84, E136, and D162, Figure 1B). Interestingly, all critically essential amino acids except K92 in CitE1 were conserved among *M. tuberculosis* CitE protein homologs (Figure 1C).

### Biochemical characterization of CitE1 and CitE2 enzymes

**Figure 2:**
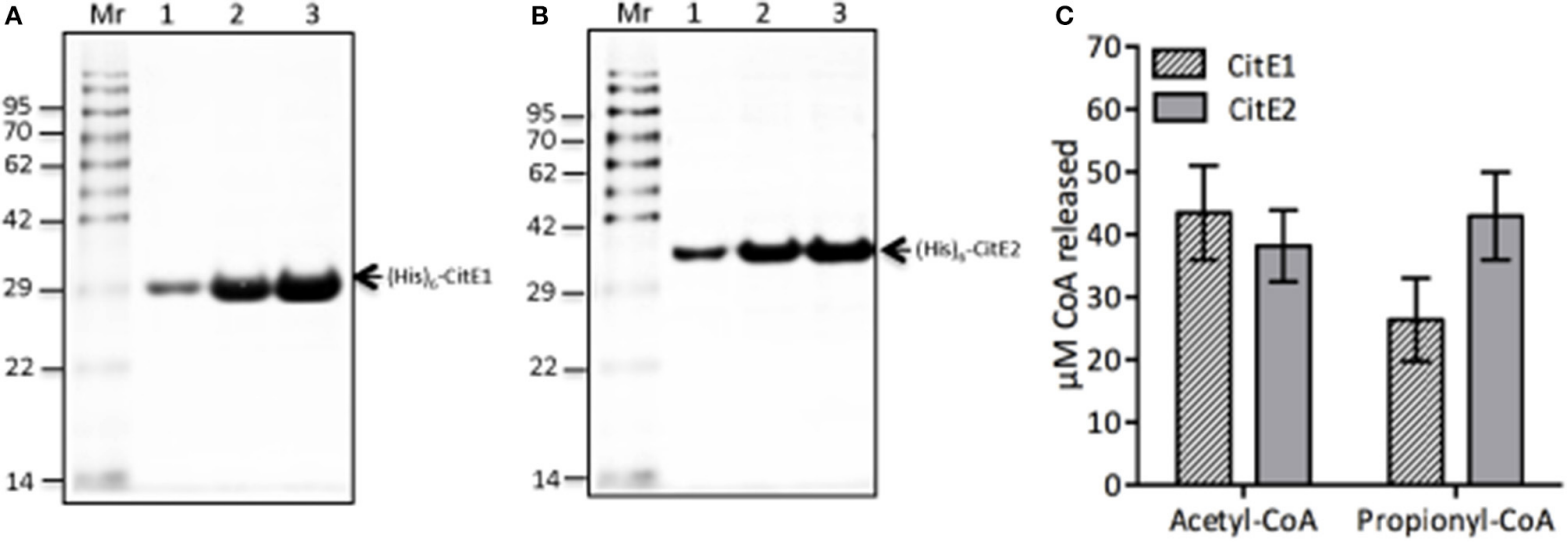
**(A,B)** Expression and purification of (His)_6_-CitE1 and (His)_6_-CitE2. The recombinant proteins (His)_6_-CitE1 (A) and (His)_6_-CitE2 **(B)** were expressed and purified using Ni^2+^-NTA affinity chromatography. Loading pattern: **(A)** Un-Whole cell lysates of uninduced *E. coli*; In-Whole cell lysates of IPTG induced cells; Lanes (1–4)-Purified fractions of (His)_6_-CitE1. **(B)** Un-Whole cell lysates of uninduced *E. coli*; In-Whole cell lysates of IPTG induced *E. coli*; Lanes (1–5)- Purified fractions of (His)_6_-CitE2. **(C)** Biochemical characterization of (His)_6_-CitE1 and (His)_6_-CitE2. Both (His)_6_-CitE1 and (His)_6_-CitE2 exhibited acetyl-CoA lyase and propionyl-CoA lyase activities. The amount of CoA released in enzymatic reaction was quantified using DTNB reagent as described in Materials and Methods. The data shown in this panel is mean ± S.E. of CoA release obtained in enzymatic reactions from three independent experiments.

CLYBL, annotated as “citrate lyase beta like” shares sequence identity of ~30% with *M. tuberculosis* CitE proteins and possesses both malate synthase and β-methyl malate synthase activity (Strittmatter et al., 2014; Supplementary Figure 2). For biochemical characterization, both homologs, CitE1 and CitE2 were expressed and purified as (His)_6_-tagged fusion proteins. We observed that both (His)_6_-CitE1 and (His)_6_-CitE2 migrated at their expected molecular mass of 29.5 and 33.5 kDa, respectively (Figures 2A,B). The purified fractions were dialyzed, concentrated and stored in buffer containing 50 mM sodium phosphate, pH 7.4, 100 mM NaCl and 10% glycerol at −80°C till further use. We next determined whether these proteins possessed either citrate lyase or malate synthase or β-methyl malate synthase activity. As expected, CitE1 and CitE2 proteins did not possess citrate lyase activity (data not shown). In contrast to CLYBL enzyme, we did not observe either malate synthase or β-methyl malate synthase activity to be associated with either CitE1 or CitE2 protein (data not shown). As positive controls both malate synthase and β-methyl malate synthase activities were detected in *M. tuberculosis* lysates from early-log phase cultures. We observed ~200 and 80 μM CoA was released in malate synthase and β-methyl malate synthase activity reactions, respectively, using 10 μg of lysates (data not shown). Intriguingly, both CitE1 and CitE2 proteins displayed non-specific CoA lyase activity (Figure 2C). In reactions containing either acetyl-CoA or propionyl-CoA, ~26–43 μM CoA release was observed after incubation for 10 min (Figure 2C).

### CitE1 and CitE2 are differentially expressed upon exposure of *M. tuberculosis* to stress conditions

**Figure 3:**
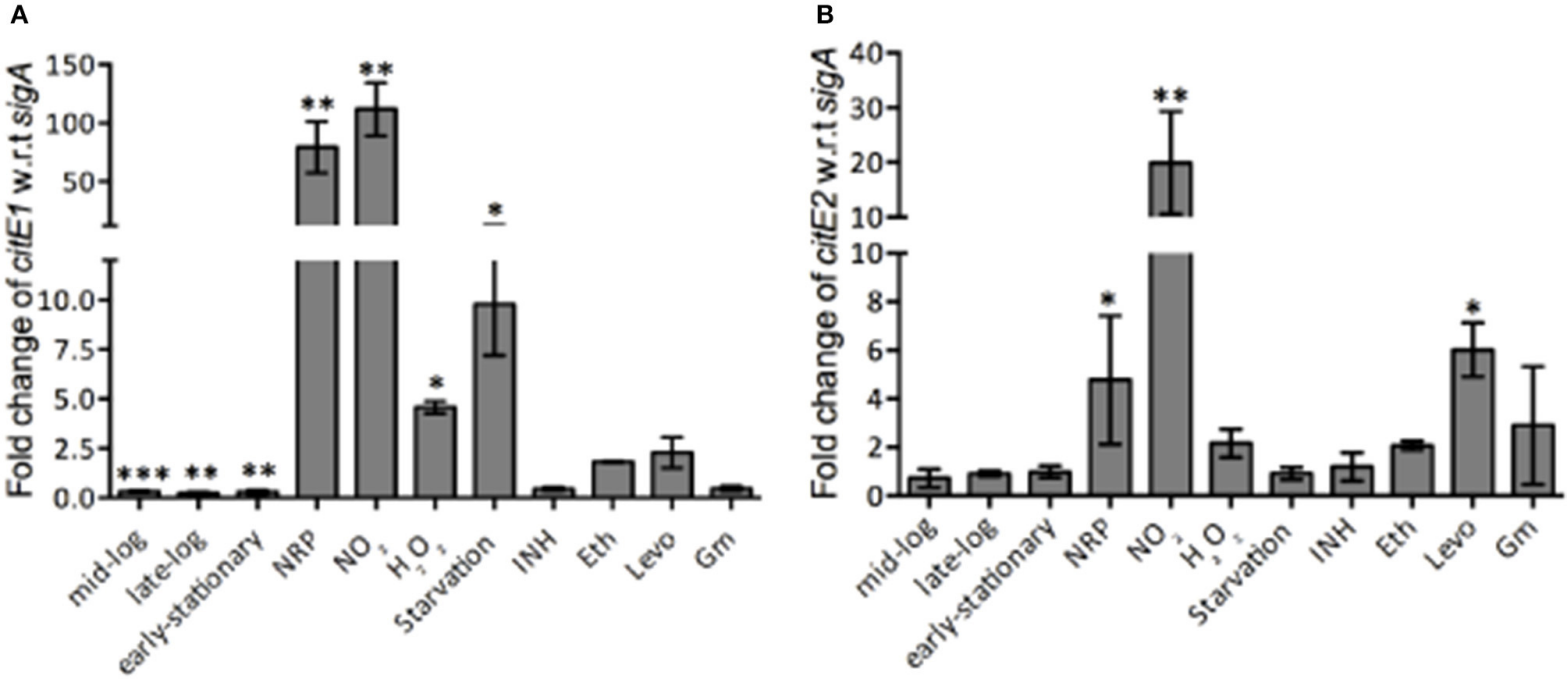
qRT-PCR analysis to measure *citE1* and *citE2* transcript levels in different conditions. qRT-PCR was performed and the relative expression levels of *citE1* **(A)** and *citE2* **(B)** was quantified after normalization to levels of *sigA* in different growth stages and and upon exposure to different stress conditions or drugs as described in Materials and Methods. The data shown in this panel is mean ± S.E. of fold change obtained from three independent experiments. Significant differences were observed for the indicated groups (paired two-tailed *t*-test, *p < 0.05, **p < 0.01, ***p < 0.001).

*M. tuberculosis* modulates the expression of various genes involved in metabolic pathways to enhance its adaptation and survival in the hostile environment of the macrophages (Betts et al., 2002; Fisher et al., 2002; Ohno et al., 2003; Voskuil et al., 2003, 2004, 2011; Flentie et al., 2016). We next quantified *citE1* and *citE2* transcript levels in *M. tuberculosis* exposed to different stress conditions that it might encounter in the host. As shown in Figures 3A,B, *citE1* transcript levels decreased by ~2.0-fold, at later stages of growth, whereas the transcript levels of *citE2* remained unaltered in these conditions. The exposure to low oxygen conditions resulted in ~60.0- and 5.0-fold increase in the transcript levels of *citE1* and *citE2*, respectively (Figures 3A,B, ^**^p < 0.01 and ^*^p < 0.05). We also noticed that *citE1* and *citE2* transcript levels were increased by 100.0- and 20.0-fold, respectively in *M. tuberculosis* upon exposure to nitrosative stress (Figures 3A,B, ^**^p < 0.01). In concordance, the transcript levels for *citE1* and *citE2* were increased by 75.0- and 4.0-fold in *M. bovis* BCG upon exposure to nitrosative stress (data not shown). This observed upregulation of *citE1* and *citE2* transcript levels was because of generation of reactive nitrogen intermediates (RNI), as these were only upregulated by 2.0- to 3.0-fold upon exposure to acidic stress (data not shown). Further, *citE1* transcript levels were upregulated by 4.0- and 10.0-fold, respectively upon *M. tuberculosis* exposure to oxidative or nutritional stress (Figure 3A, ^*^p < 0.05). As shown in Figure 3B, *citE2* transcript levels remained unchanged in these stress conditions.

Bacterial persistence relies on metabolic pathways to establish infection and persist in the host. For example, isocitrate lyase, in addition to its role in glyoxylate shunt and methyl citrate cycle, also protects *M. tuberculosis* from the oxidative stress of isoniazid, rifampicin and streptomycin *in vitro* (Nandakumar et al., 2014). We next determined the transcript levels of both *citE1* and *citE2* in *M. tuberculosis* exposed to either inhibitor of cell wall (isoniazid or ethambutol) or replication (levofloxacin) or translation (gentamycin). We observed that the transcript levels of *citE1* were downregulated by ~2.0-fold in *M. tuberculosis* upon exposure to either isoniazid or gentamycin (Figure 3A). However, exposure to ethambutol resulted in 2.0-fold increase in the transcript levels of both *citE1* and *citE2* (Figures 3A,B). As shown in Figures 3A,B, during *M. tuberculosis* exposure to levofloxacin, *citE1* transcript levels remained unchanged, while *citE2* transcript levels were upregulated by ~6.0-fold in these conditions (^*^p < 0.05).

### Construction and characterization of various single and double mutant strains of *M. tuberculosis*

In order to investigate the role of CitE1 and CitE2 in *M. tuberculosis* stress adaptation, physiology and pathogenesis, single and double mutant strains of *M. tuberculosis* were generated using temperature-sensitive mycobacteriophages as described in Materials and Methods. In the Δ*citE1* and Δ*citE2* single mutant strain, the open reading frame for Rv2498c and Rv3075c were replaced with hygromycin resistance gene, respectively (Supplementary Figure 3A). In double mutant, Δ*citE-DM* strain, the *citE2* open reading frame was replaced with kanamycin resistance gene in the genome of Δ*citE1* strain (Supplementary Figure 3A). The replacement of *citE1* and *citE2* with antibiotic-resistance genes in their respective mutant strains was confirmed by PCR using locus-specific primers (Supplementary Figure 3B). As expected, *citE1* locus specific primers yielded PCR amplicon of 1.2 kb in the case of parental strain and 2.2 kb in the case of Δ*citE1* and Δ*citE-DM M. tuberculosis* strains (Supplementary Figure 3B). PCR amplification using *citE2* locus primers yielded amplicons of sizes 1.1, 1.9, and 1.7 kb in the case of wild type, Δ*citE2* and Δ*citE-DM M. tuberculosis* strains, respectively (Supplementary Figure 3B). The construction of various mutant strains in *M. tuberculosis* was also confirmed by Southern blot analysis using locus specific primers (Supplementary Figure 3C). In order to rule out the possibility that disruption of *citE1* and *citE2* had any polar effect on the transcription of neighboring genes, we quantified their transcript levels in both wild type and Δ*citE-DM* strain. We observed that the transcript levels of Rv2497c, Rv2499c, Rv3074, and Rv3076 remained unchanged in wild type and Δ*citE-DM* strain (Supplementary Figure 3D).

### CitE enzymes are essential for growth of *M. tuberculosis* on cholesterol containing medium

**Figure 4:**
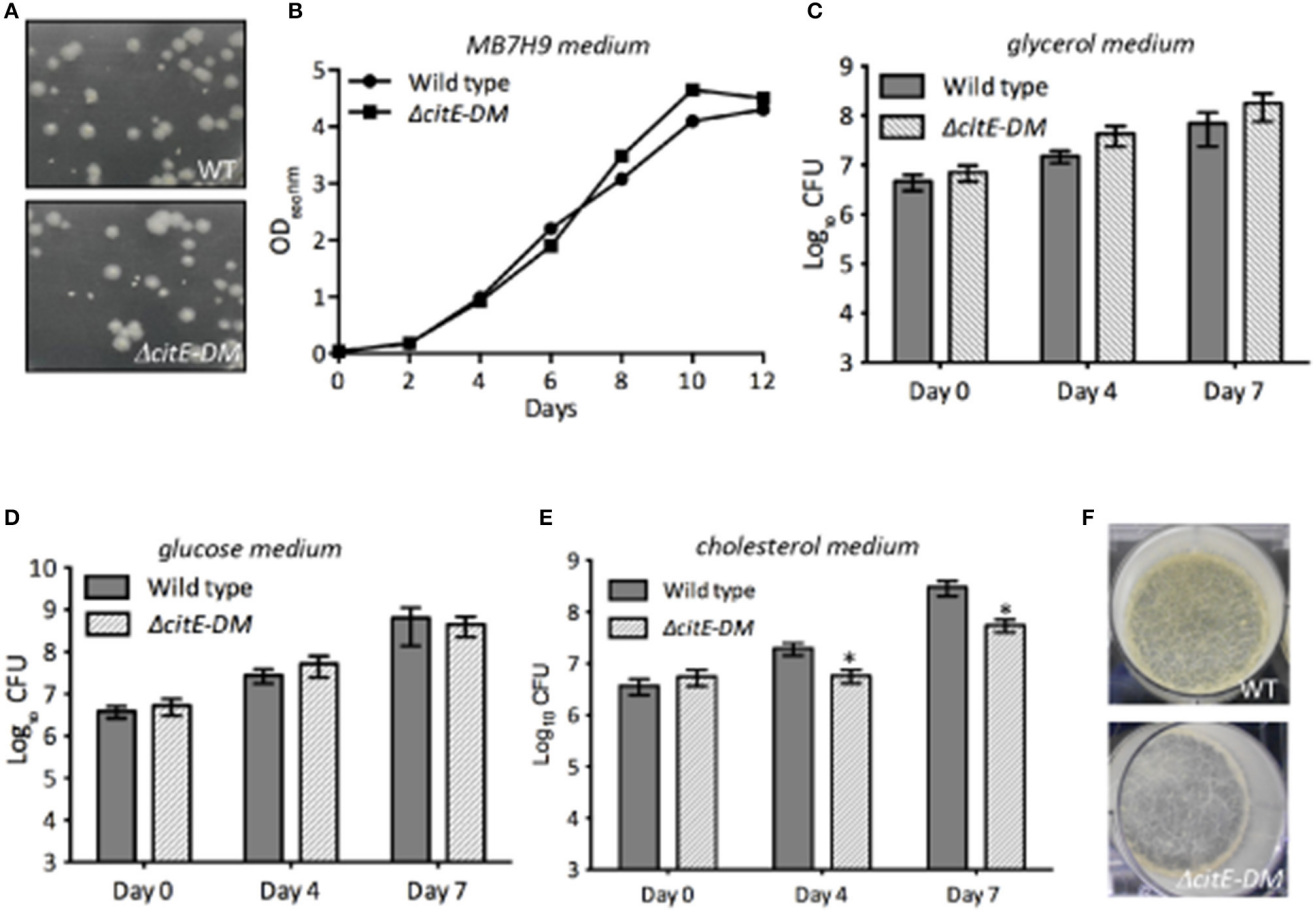
Influence of the deletion of *citE1* and *citE2* on *M. tuberculosis* growth. **(A)** The colony morphology of wild type and Δ*citE-DM* strain of *M. tuberculosis* was determined by plating 10-fold serial dilution of mid-log phase grown cultures on MB7H11 plates at 37°C for 3–4 weeks. **(B)** The growth patterns of wild type and Δ*citE-DM* strains in MB7H9 medium was monitored by determining OD_600nm_. **(C–E)** The bacterial loads of *M. tuberculosis* wild-type and Δ*citE-DM* mutant strain were determined at the time of inoculation, day 4 and 7 of growth in Sauton's medium containing either 0.4% glycerol **(C)** or 0.4% glucose **(D)** or 0.01% cholesterol **(E)**. The data shown in this panel is mean ± S.E. of bacterial loads obtained from three independent experiments. Significant differences were observed for the indicated groups (paired two-tailed *t*-test, *p < 0.05). **(F)** Biofilm images of the wild type and Δ*citE-DM* strains. For biofilm formation, various strains were grown in Sauton's medium without tween-80 in 6 well plates (without shaking) at 37°C for 3–4 weeks.

In order to understand the role of CitE enzymes in *M. tuberculosis* growth *in vitro*, we compared the growth characteristics of parental and double mutant strain *in vitro*. As shown in Figure 4A, the colony morphology of both strains was similar on solid medium. The growth of both strains was also comparable in MB7H9 medium (Figure 4B). To further study the effect of CitE enzymes in metabolism, we compared the ability of wild type and Δ*citE-DM* strain to grow in Sauton's medium containing either glucose or glycerol or cholesterol as carbon source. As shown in Figures 4C,D no significant differences were seen in the growth patterns of parental and double mutant strain in either glycerol or glucose containing medium. Inefficient metabolism of cholesterol results in propionyl-CoA accumulation and this accumulation is toxic for *M. tuberculosis* (Muñoz-Elías et al., 2006). In concordance with our biochemical assays, double mutant strain displayed a slight growth defect of ~3.0- to 5.0-fold in Sauton's medium containing cholesterol (Figure 4E, ^*^p < 0.05). However, we did not observe any significant differences in the growth patterns of wild type, Δ*citE*1 and Δ*citE*2 strains in cholesterol containing medium (Supplementary Figure 4A). These findings demonstrate that these enzymes are cumulatively required for growth in cholesterol containing medium. We also observed that simultaneous deletion of CitE1 and CitE2 enzymes in *M. tuberculosis* genome did not impair its ability to form biofilms *in vitro* (Figure 4F).

### Inactivation of CitE enzymes in *M. tuberculosis* increases sensitivity to oxidative and detergent stress

**Figure 5:**
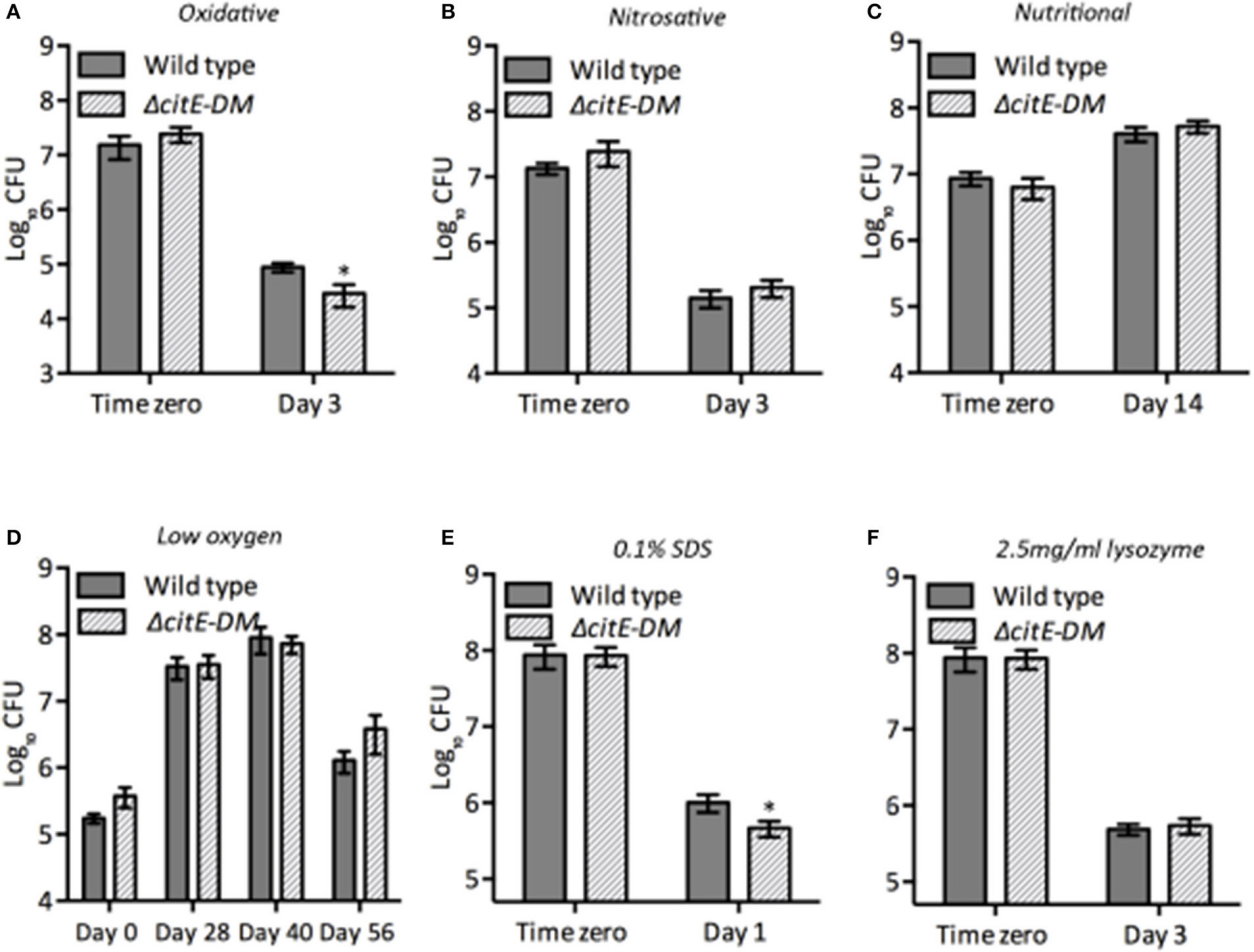
Susceptibility of various strains to different stress conditions. Early-log phase cultures of wild type and Δ*citE-DM* strains were exposed to either oxidative **(A)** or nitrosative **(B)** or nutritional **(C)** or low oxygen **(D)** or 0.1% SDS **(E)** or 2.5 mg/ml lysozyme **(F)** as described in Materials and Methods. At designated time points 10.0-fold serial dilutions were prepared and 100 μl was plated on MB7H11 plates and incubated at 37°C for 3–4 weeks. The data shown in this panel is mean ± S.E. obtained from at least two independent experiments. Significant differences were observed for the indicated groups (paired two-tailed *t*-test, *p < 0.05).

*M. tuberculosis* is a highly successful intracellular pathogen by virtue of its ability to modulate its cellular physiology and metabolism in order to adapt to the hostile conditions it encounters during infection. Various metabolic enzymes have been shown to be essential for *M. tuberculosis* adaptation to different stress conditions (Rhee et al., 2011; Cumming and Steyn, 2015). Hence, we next evaluated the contribution of CitE enzymes in adaptation of *M. tuberculosis* to different stress conditions. We compared the survival of the parental and Δ*citE-DM* strains upon exposure to either oxidative or nitrosative or nutritional or low-oxygen or lysozyme or detergent stress (Figure 5). In comparison to parental strain, double mutant strain was more susceptible to killing by ~5.0-fold upon exposure to oxidative stress conditions (Figure 5A, ^*^p < 0.05). In concordance with our earlier findings, we observed that parental, Δ*citE1* and Δ*citE2* single mutant strains survived at comparable levels upon exposure to oxidative stress conditions (Supplementary Figure 4B). We observed that in comparison to the parental strain, double mutant strain exhibited a 4.0-fold decrease in bacterial counts upon exposure to 0.1% sodium dodecyl sulfate (SDS, Figure 5E). The double mutant strain, Δ*citE-DM* survived at rates comparable to those observed for wild type strain upon exposure to other stress conditions tested (Figures 5B–D,F). These observations implicate that deletion of CitE enzymes increases sensitivity of *M. tuberculosis* upon exposure to either oxidative or detergent stress.

### CitE enzymes are required for optimal growth in macrophages

**Figure 6:**
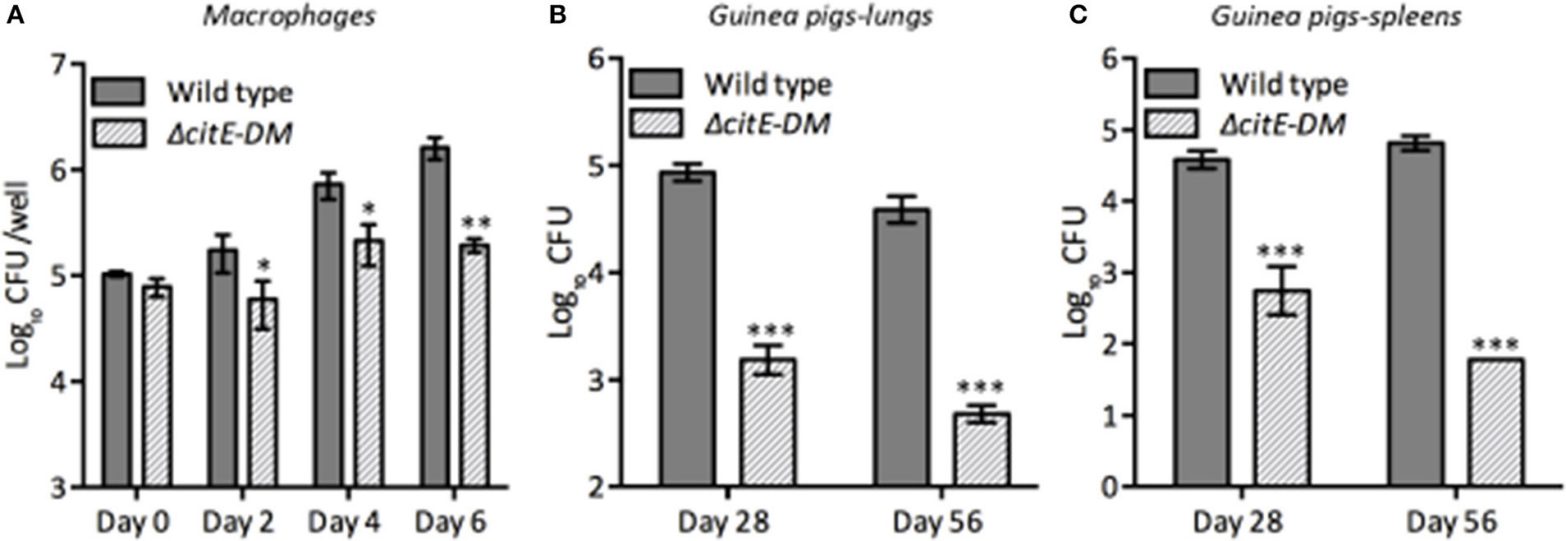
Influence of deletion of the *citE1* and *citE2* genes on the growth and survival of *M. tuberculosis* in macrophages and *in vivo*: **(A)** THP-1 monocytes were differentiated and infected with various strains. The number of intracellular viable bacteria was determined at day 0, 2, 4, and 6 post-infection. The data shown in this panel is mean ± S.E. of bacterial loads obtained from three independent experiments. **(B,C)** Female guinea pigs were infected with various strains via aerosol route and bacterial loads were determined in lungs **(B)** and spleens **(C)** at either 4 or 8 weeks post-infection. The data is depicted as mean ± S.E. of bacterial loads obtained from 6 or 7 animals per time point. Significant differences were observed for the indicated groups (paired two-tailed *t*-test, *p < 0.05, **p < 0.01 and ***p < 0.001).

*M. tuberculosis* is an intracellular pathogen and macrophages provide the first line of defense in the lungs in response to *M. tuberculosis* infection (McDonough et al., 1993). *M. tuberculosis* by virtue of its metabolic flexibility can persist and replicate within macrophages (Rhee et al., 2011; Warner, 2014; Eoh et al., 2017). We next determined whether simultaneous disruption of *citE1* and *citE2* impairs ability of *M. tuberculosis* to grow within human macrophages. We compared the growth kinetics of parental and double mutant strain in THP-1 macrophages at day 2, 4, and 6 post-infection. As shown in Figure 6A, intracellular bacterial counts were comparable for both strains at day 0 post-infection. However, at 4 days post-infection, the viable counts in macrophages infected with the double mutant strain was reduced by 4.0-fold in comparison to parental strain infected macrophages (Figure 6A, ^*^p < 0.05). These differences in bacterial counts of the double mutant and wild type strain increased to 10.0-fold at 6 days post-infection (Figure 6A, ^**^p < 0.01). We also observed that both CitE1 and CitE2 enzymes are individually dispensable for growth of *M. tuberculosis* in macrophages (Supplementary Figure 4C). These results demonstrated that simultaneous deletion of CitE1 and CitE2 rendered *M. tuberculosis* incapable of growing in THP-1 macrophages.

### CitE enzymes are required for *in vivo* pathogenesis in guinea pigs

To test the essentiality of CitE enzymes in the metabolism of *M. tuberculosis* during *in vivo* infection, guinea pigs were infected with either parental or double mutant strain. Guinea pigs are highly sensitive to TB infection and develop well-structured granulomas with central necrosis, a pathology similar to that observed in humans (McMurray, 1994). The bacterial loads in lungs of wild type and Δ*citE-DM* infected guinea pigs were comparable at day 1 post-infection (data not shown). However, Δ*citE-DM* was severely attenuated for growth in guinea pigs at both 4 and 8 weeks post-infection. The bacterial loads were lower by 75.0- to 90.0-folds in lungs and spleens of Δ*citE-DM* infected guinea pigs in comparison to guinea pigs infected with the parental strain at 4 weeks post-infection (Figures 6B,C, ^***^p < 0.001). This difference in bacterial loads increased to 90.0- and 1000.0-folds in lungs and spleens, respectively, at 8 weeks post-infection (Figures 6B,C, ^***^p < 0.001). The bacillary loads in lungs and spleens of 8-weeks wild type infected guinea pigs were 4.5 log_10_ CFU and 4.8 log_10_ CFU, respectively (Figures 6B,C). In contrast, the bacillary loads in lungs and spleens of Δ*citE-DM* infected guinea pigs were 2.8 log_10_ CFU and 1.7 log_10_ CFU, respectively at 8 weeks post-infection (Figures 6B,C). These observations show that CitE enzymes contribute cumulatively to the ability of *M. tuberculosis* to replicate and cause infection in guinea pigs.

**Figure 7:**
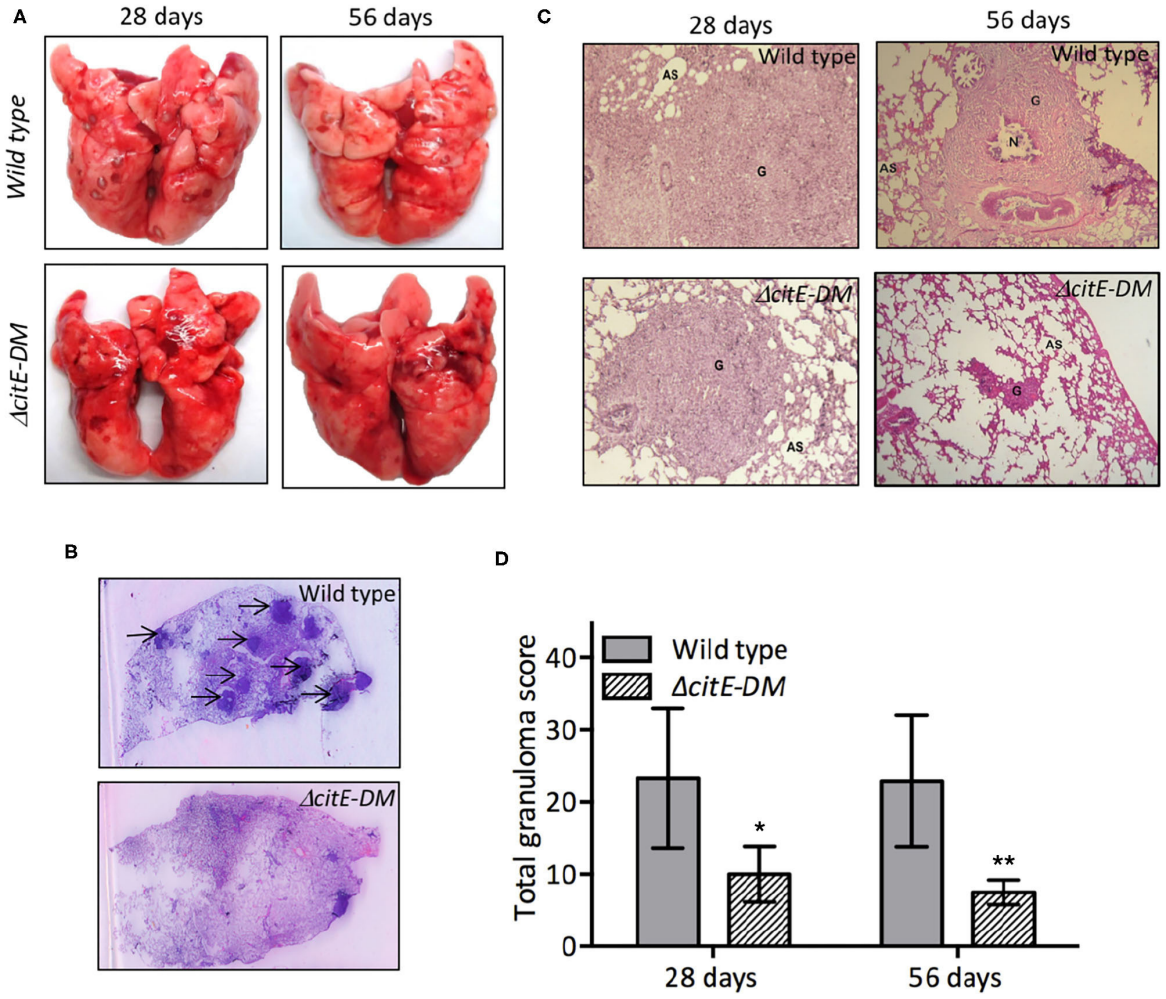
Gross pathological and histopathological analysis of lungs of infected guinea pigs. **(A)** This panel depicts representative photographs of lungs showing granulomatous lesions from guinea pigs infected with various strains at 4 or 8 weeks post-infection. **(B)** High-resolution scans (2,400 dpi) of lung sections from infected guinea pigs were performed at 8 weeks post-infection. A representative high-resolution photomicrograph for each group is shown and granulomas are marked by arrows**. (C)** Images of H & E stained lung sections from guinea pigs at day 28 and 56 post-infection. These images were taken at 40× magnification and show granulomas (G), areas of necrosis (N) and alveolar spaces (AS). **(D)** Total granuloma score in H&E-stained lung sections of animals infected with wild type or Δ*citE-DM* at both 4 and 8 weeks post-infection. Significant differences were observed for the indicated groups (paired two-tailed *t*-test, *p < 0.05, **p < 0.01).

We also compared the tissue pathology of lung and liver sections from wild type and Δ*citE-DM* strain infected guinea pigs. In concordance with bacterial counts, we observed decreased gross pathology in lungs from Δ*citE-DM* infected guinea pigs in comparison to parental strain infected guinea pigs at both 4 and 8 weeks post-infection (Figure 7A). The lung tissues from parental strain infected guinea pigs exhibited heavy tissue involvement with numerous tubercles (Figure 7A). In contrast, lungs from Δ*citE-DM* infected guinea pigs exhibited reduced pathology with minimum involvement (Figure 7A). In concordance, spleens from parental strain infected guinea pigs displayed splenomegaly with several large tubercles, whereas no tubercles were observed in spleens from Δ*citE-DM* infected guinea pigs (data not shown). High resolution scanning images showed more number of granulomas in sections from wild type infected guinea pigs in comparison to sections from mutant strain infected guinea pigs (Figure 7B). In our histopathology analysis, we observed lesser tissue damage and reduced granuloma formation in sections from Δ*citE-DM* infected guinea pigs at 4 and 8 weeks post-infection (Figure 7C). As shown in Figure 7C, severe granulomatous inflammation and loss of parenchymal space was observed in sections from parental strain infected guinea pigs at both 4 and 8 weeks post-infection. The detailed analysis of tissue damage in H & E stained sections revealed that total granuloma score in lungs and liver sections from parental strain infected guinea pigs was 23.0 and 22.1, respectively, at 8 weeks post-infection (Figure 7D, data not shown). In comparison, the total granuloma score in lungs and liver of double mutant infected guinea pigs was 7.5 and 0.0, respectively at 8 weeks post-infection (Figure 7D, data not shown, ^**^p < 0.01). Taken together, these observations suggest that CitE enzymes are indispensable for *M. tuberculosis* pathogenesis *in vivo*.

### Inactivation of CitE1 and CitE2 does not alter lipid profile or redox status of *M. tuberculosis*

The mycobacterial cell envelope is lipid rich and lipids, such as trehalose containing glycolipids, lipoglycans derived from phosphatidylinositol and those containing mycocerosate are essential for *M. tuberculosis* pathogenesis (Neyrolles and Guilhot, 2011). Further, lipids provide structural integrity and act as a permeability barrier against drugs and other toxic molecules. It was earlier suggested that *M. tuberculosis* CitE1 might play an essential role in fatty acid metabolism (Goulding et al., 2007). Since Δ*citE-DM* mutant strain was attenuated for growth in guinea pigs, we hypothesized that this might be attributed to altered lipid profiles. To test this hypothesis, we compared the lipid profiles of mid-log phase cultures of wild type and Δ*citE-DM* strains. For lipid analysis, apolar and polar lipid fractions were extracted and resolved on 2D-TLC using different solvent systems as described in Materials and Methods. We did not observe any significant difference in the profiles of both polar and apolar lipids isolated from wild type and double mutant strains. The spots corresponding to phthiocerol dimycocerosate (PDIM), di- and tri- acyl glycerol (TAG) as well as free mycolic acids (FMA) were observed in apolar lipids from both strains (Supplementary Figure 5). The levels of diacyl trehalose (DAT) and phosphatidylinositol mannosides (PIMs) in polar lipid fractions isolated from wild type and Δ*citE-DM* strain were comparable (Supplementary Figure 5). In addition to the lipid profiles, we also compared the ratios of NADH/NAD^+^ in both strains at different stages of growth. We observed that these ratios were comparable between these strains and varied between 0.5 and 1.0 at different stages of growth (data not shown).

## Discussion

Citrate lyase enzymes are oligomeric comprising of three subunits, α-, β-, and γ-. In *P. fluorescens*, citrate lyase enzyme is involved in metabolic reprogramming for anaerobic utilization of citrate to counter nitrosative stress conditions (Auger and Appanna, 2015). In photosynthetic anaerobic bacteria, such as *Chlorobium limicola*, citrate lyase has been shown to be responsible for CO_2_ assimilation in anaerobic conditions (Ivanovsky et al., 1980; Antranikian et al., 1982). The genome of various bacterial pathogens encodes only for β-subunit of citrate lyase, thereby suggesting an unconventional role for these enzymes. For example RipC, an annotated citrate lyase β-subunit from *Yersinia pestis* has been hypothesized to possess CoA binding activity and might be involved in virulence (Torres et al., 2012). *M. tuberculosis* genome lacks α- and γ-subunits of citrate lyase but encodes for two homologs of β-subunits of citrate lyase. The structure of Rv2498c (CitE1) has been solved and all residues important for catalysis are well conserved in *M. tuberculosis citE* homologs (Goulding et al., 2007). Here, we sought to investigate the role of CitE enzymes in *M. tuberculosis* physiology and pathogenesis.

CLYBL, a homolog of β-subunit of citrate lyase possesses dual malate synthase and β-methyl malate synthase activity (Strittmatter et al., 2014). However, *M. tuberculosis* CitE homologs lacked malate synthase or β-methyl malate synthase activity, but were able to non-specifically degrade acetyl-CoA or propionyl-CoA. These observations implicate that these enzymes might be involved in detoxification of propionyl-CoA that accumulates in bacteria upon metabolism of β-oxidation of odd chain fatty acids. The expression of these enzymes was upregulated in stress conditions, such as nitrosative and low oxygen conditions, thereby indicating that these enzymes contribute to *M. tuberculosis* adaptation in these conditions. In concordance with the essentiality predictions for these proteins, we were able to generate both single and double *citE* mutant strains of *M. tuberculosis* (Sassetti et al., 2003; Griffin et al., 2011). As expected, deletion of both *citE1* and *citE2* did not alter colony morphology or growth kinetics of *M. tuberculosis* in medium containing either glucose or glycerol as sole carbon source. The major source of carbon utilized by *M. tuberculosis in vivo* is lipids and interestingly, double mutant strain exhibited a slight growth defect in cholesterol containing medium. We also noticed that simultaneous deletion of *citE1* and *citE2* from *M. tuberculosis* genome rendered the bacteria more sensitive to oxidative stress. In order to counteract ROI production in host, *M. tuberculosis* has developed various defense mechanisms, such as production of cell wall associated lipids, secretory antioxidant enzymes, redox buffers, such as ergothionine, mycothiols, and thioredoxins (Yuan et al., 1995; Murry et al., 2009; Kumar et al., 2011). In order to further delineate the downstream effects of *citE1* and *citE2* deletion from *M. tuberculosis* genome, lipid profiles and intracellular redox ratios of mutant and wild-type strains were compared. We observed that the lipid profiles and redox ratios of these strains were comparable, thereby, suggesting that these proteins neither contribute toward lipid architecture nor redox homeostasis.

Following inhalation by the host, *M. tuberculosis* enters airways and alveolar macrophages provide the first line of defense against infection. The double mutant strain showed impaired survival inside macrophages, thereby, suggesting that these enzymes are essential for intracellular survival of *M. tuberculosis*. Similarly, *citE* mutant strain of *Cryptococcus neoformans* was also impaired for survival in macrophages (Griffiths et al., 2012). In concordance, the double mutant strain was also attenuated for growth and survival in guinea pigs *in vivo*. This observed growth defect might be attributed to requirement of these enzymes to counteract oxidative stress encountered by *M. tuberculosis* inside host. In concordance, key enzymes belonging to CCM, such as DlaT, FGD1 and fumarase that are required for tolerance to oxidative stress are also essential for *M. tuberculosis* pathogenesis (Shi and Ehrt, 2006; Gurumurthy et al., 2013; Ruecker et al., 2017). *M. tuberculosis* triggers alterations in various metabolic pathways which enables the bacteria to utilize different carbon sources, such as sugars and fatty acids to generate energy. Since, CitE enzymes non-specifically degrades propionyl-CoA, there is a possibility that this growth defect associated with the double mutant *in vivo* might be due to accumulation of toxic propionyl-CoA. Similarly, *M. tuberculosis* strains with a defect in either cholesterol catabolism or uptake are also attenuated for growth in both macrophages and mice tissues (Pandey and Sassetti, 2008; Mdluli et al., 2014). These findings suggest that *M. tuberculosis* remodels central carbon metabolic pathways to ensure its survival in the hostile environment encountered *in vivo*.

In conclusion, these findings imply that these enzymes are essential for *M. tuberculosis* to establish infection in the host. Future studies would include experiments to understand the mechanisms by which these enzymes contribute to *M. tuberculosis* virulence and to identify small molecule inhibitors against these enzymes. Validating these unexplored enzymes as drug target would be of great significance as anti-tubercular drugs with novel targets are crucial in the fight for the global eradication of TB.

## Materials and methods

### Ethics statement

The animal care and protocols used in the present study adhered to the guidelines provided by Committee for the Purpose of Control and Supervision of Experiments on Animals (CPCSEA), Government of India. The animal protocols were reviewed and approved by the Institutional Animal Ethics Committee of International Center for Genetic Engineering and Biotechnology, New Delhi (ICGEB, New Delhi, ICGEB/AH/2015/TACF/THSTI-03).

### Bacterial strains and culture conditions

The plasmids and strains used in the present study are listed in Table 1. Most of the chemicals used unless mentioned were procured from Sigma-Aldrich. *E. coli* strains, XL-1 Blue and HB-101 were used for cloning purpose. BL-21 (λDE3, pLysS) strain was used for protein expression and purification studies. The culturing of *E. coli* was performed in Luria–Bertani broth or Luria–Bertani agar as per standard protocols. The culturing of mycobacterial strains in both liquid and solid medium was performed as previously described (Singh et al., 2013). For carbon source experiments, *M. tuberculosis* strains were cultured in Sauton's medium (0.5 g/L asparagine, 1.0 g/L KH2PO_4_, 2.5 g/L Na2HPO_4_, 50 mg/L ferric ammonium citrate, 0.05g/L MgSO_4_·7H_2_0, 0.05 g/L CaCl_2_, 0.01 mg/L ZnSO_4_ and 0.05% Tyloxapol) containing either 0.4% v/v glycerol or 0.4% v/v glucose or 0.01% v/v cholesterol. The antibiotics were used at the following concentrations; ampicillin (100 μg/ml for *E. coli*), kanamycin (50 μg/ml for *E. coli* and 25 μg/ml for mycobacteria), tetracycline (10 μg/ml for *E. coli*), chloramphenicol (34 μg/ml for *E. coli*) and hygromycin B (150 μg/ml for *E. coli* and 50 μg/ml for mycobacteria).

**Table 1:** List of strains and plasmids used in the study.

**Strains**	**Description**	**References**
XL-1 Blue	*recA1 endA1 gyrA96 thi-1 hsdR17 supE44 relA1 lac [F′ proAB lacIq ZΔM15 Tn10 (Tetr)]*	Stratagene, USA
HB-101	*F-, thi-1, hsdS20 (r_*B*_- m_*B*_), supE44, recA13, ara-14, leuB6, proA2, lacY1, galK2, rpsL20 (strr), xyl-5, mtl-1*	Promega, USA
BL-21(λDE3), plysS	*F-, ompT, hsdS _*B*_ (r_*B*_-m_*B*_), dcm, gal, λ(DE3), plysS, cmr*	Promega, USA
*mc^2^155*	*M. smegmatis* parental strain	A kind gift from Prof. Anil K Tyagi.
H_37_Rv	Laboratory Strain (ATCC 27294) of *M. tuberculosis*	ATCC
*M. bovis* BCG Danish	*M. bovis* BCG parental strain	A kind gift from Prof. Anil K Tyagi.
Δ*citE1*	*citE1* mutant strain of *M. tuberculosis*	This study
Δ*citE2*	*citE1* mutant strain of *M. tuberculosis*	This study
Δ*citE-*DM	*citE1* and *citE2* double deletion strain of *M. tuberculosis*	This study
**PLASMIDS**		
pYUB854	Cloning vector, hyg^R^	Bardarov et al., 2002
pYUB-Δ*citE1*	pYUB854 vector carrying upstream and downstream regions of *citE1*, hygromycin cassette	This study
pYUB-Δ*citE2*	pYUB854 vector carrying upstream and downstream regions of *citE2*, hygromycin cassette	This study
pYUB-Δ*citE2 kan*	pYUB854 vector carrying upstream and downstream regions of *citE2*, kanamycin cassette	This study
phAE87	Phagemid DNA, Amp^R^	Bardarov et al., 2002
phAE87-Δ*citE1*	Phagemid DNA carrying upstream and downstream regions of *citE1*, hygromycin cassette	This study
phAE87-Δ*citE2*	Phagemid DNA carrying upstream and downstream regions of *citE2*, hygromycin cassette	This study
phAE87-Δ*citE2 kan*	Phagemid DNA carrying upstream and downstream regions of *citE2*, kanamycin cassette	This study
pET28b	*E. coli* T7-based expression system	Novagen
pET-28b-*citE1*	pET28b carrying *citE1*	This work
pET-28b-*citE2*	pET28b carrying *citE2*	This work

### Multiple sequence alignment and construction of homology model

For alignment studies, protein sequences of CitE enzymes from various microorganisms were retrieved from the National Center for Biotechnology Information (NCBI) protein database. Multiple sequence alignment analysis among these proteins was performed using Clustal Omega (version 1.2.0) alignment tool and edited using GeneDoc. The construction of phylogenetic tree and analysis of evolutionary history was conducted in MEGA7 software using the Minimum Evolution method (Kumar et al., 2016).

### Protein purification and biochemical characterization of CitE1 and CitE2 enzymes

The sequences of oligonucleotides used in this study are shown in Supplementary Table 1. For biochemical characterization, genes encoding either CitE1 or CitE2 were PCR amplified, sequenced and cloned into pET28b resulting in pET28b-*citE1* and pET28b-*citE2*, respectively. The recombinant plasmids were transformed into *E. coli* BL-21 (λDE3, pLysS) and expression of recombinant proteins was induced by overnight addition of 1 mM IPTG at 18°C. (His)_6_-CitE1 and (His)_6_-CitE2 were purified from cytosolic fractions and purity of fractions was assessed on 12.5% SDS PAGE. The purified fractions were pooled, dialyzed and concentrated in buffer containing 20 mM sodium phosphate, pH-7.4, 200 mM NaCl and 10% glycerol. The purified CitE1 and CitE2 proteins were assayed for either citrate lyase or malate synthase or β-methyl malate synthase activity. Citrate lyase activity was assayed using malate dehydrogenase coupled assay and reaction was monitored by measuring absorbance at 340 nm (Srere, 1963). The malate synthase and β-methyl malate synthase activity was performed in buffer containing 100 mM Tris pH-8.0, 10 mM glyoxylate with either 1 mM acetyl-CoA or propionyl CoA, respectively (Strittmatter et al., 2014). The amount of CoA released in these enzymatic reactions was determined by the addition of 5,5′-dithio-bis-[2-nitrobenzoic acid] (DTNB) reagent and measuring absorbance at 412 nm. All enzymatic assays included their respective buffer only, no enzyme and no substrate controls. The absorbance change in enzymatic reactions was calculated using the following formula ΔA_630 nm_ = Abs_630 nm_ of total reaction – (Abs_630 nm_ of no substrate control + Abs_630 nm_ of no enzyme control – Abs_630 nm_ of buffer only). The standard curve for CoA in the range of 10–320 μM was also prepared to calculate CoA released in our enzymatic reactions.

### RNA isolation and qPCR studies

Early-log phase cultures of *M. tuberculosis* or *M. bovis* BCG were subsequently exposed to different stress conditions or anti-mycobacterial agents as previously described (Singh et al., 2013, 2016). Total mRNA was isolated from these cultures as previously described using RNAeasy columns. For qPCR, 200 ng of DNaseI treated mRNA was subjected to cDNA synthesis using Superscript III reverse transcriptase as per manufacturer's recommendations (Thermo Fisher). The synthesized cDNA was subjected to qPCR using gene specific primers and SYBR green mix (Applied Biosystems). The data obtained was normalized to the transcript levels of housekeeping gene, *sigA* and quantified as previously described (Singh et al., 2013).

### Generation and validation of single and double mutants

In order to investigate the role of CitE enzymes in *M. tuberculosis* physiology and pathogenesis, *citE* single and double mutant strains were generated using temperature sensitive mycobacteriophages as per standard protocols (Bardarov et al., 2002). The recombinant phagemids were electroporated in *M. smegmatis* to generate high-titer temperature sensitive mycobacteriophages. For construction of mutant strains, mid-log phase cultures of *M. tuberculosis* were transduced with these mycobacteriophages and transductants were selected on MB7H11 plates containing hygromycin. The replacement of *citE1* and *citE2* genes with hygromycin resistance gene in their respective mutant strains was confirmed by PCR and Southern blot analysis. The *citE* double mutant strain of *M. tuberculosis* was constructed by replacing open reading frame for *citE2* with kanamycin resistance gene in the genome of Δ*citE1* strain. The construction of Δ*citE-DM* strain in *M. tuberculosis* was confirmed by PCR and Southern blot analysis.

### Growth kinetics and *in vitro* stress experiments

For growth kinetics, late-log phase cultures were harvested, washed twice with 1× phosphate buffered saline (PBS) and diluted to an OD_600nm_ of 0.05 in either MB7H9 medium or defined Sauton's minimal medium containing either glycerol or glucose or cholesterol as the sole carbon source. The growth patterns of parental and double mutant strain were determined by CFU analysis. The survival of parental and double mutant strain was also compared upon exposure to different stress conditions, such as nitrosative, oxidative, low oxygen, nutritional, detergent or lysozyme as per standard protocols (Singh et al., 2013, 2016). At designated time points 10.0-fold serial dilutions were prepared and 100 μl was plated on MB7H11 plates at 37°C for 3–4 weeks.

### Lipid extraction and analysis

For lipid extraction, mid-log phase cultures of various strains were harvested, resuspended in 1× PBS and heat inactivated. The extraction of apolar and polar lipids from these strains was performed as previously described (Dobson et al., 1985). The extracted apolar and polar lipids were air-dried and resuspended in either dichloromethane or chloroform:methanol (2:1), respectively. Equal amounts of lipid samples were spotted on TLC plates and resolved using different solvent systems as previously described (Dobson et al., 1985). The lipid spots were visualized by staining TLC plates with either 5% phosphomolybdic acid or 5% α-naphthol in ethanol.

### Intracellular redox ratio determination assays

For determination of intracellular redox ratios, various strains were harvested at either OD_600nm_ ~0.5 or 1.0 or 2.0 or 3.0. The bacterial pellets were resuspended in either 0.2 M HCl (for NAD^+^ extraction) or 0.2 M NaOH (for NADH extraction) and incubated at 55°C for 10 min. These solutions were subsequently neutralized by addition of 0.2 M NaOH (for NAD^+^ extraction) or 0.2 M HCl (for NADH extraction). The neutralized samples were centrifuged and levels of NAD^+^ and NADH were measured in supernatants using NAD cycling assay as previously described (Vilchèze et al., 2005).

### THP-1 macrophage infection

For macrophage experiments, THP-1 monocytes were differentiated by addition of 30 nM phorbol-12-myristate-13-acetate (PMA) and seeded at a density of 5 × 10^5^ per well. Following differentiation, macrophages were infected with various strains at a multiplicity of infection of 1:1. The number of intracellular bacteria was determined by lysing macrophages with 1× PBS-0.1% triton X-100 at day 2, 4, and 6 post-infection. For bacterial enumeration, 10.0-fold serial dilutions of lysates were prepared and 100 μl was plated on MB7H11 plates at 37°C for 3–4 weeks.

### Animal experiments

For animal experiments, pathogen-free outbred female guinea pigs of the Duncan-Hartley strain (weight, 200–300 g) were purchased from Lala Lajpat Rai University of Veterinary and Animal Sciences, Hisar, India. The infection experiments were performed at the Tuberculosis Aerosol Challenge Facility, International Center for Genetic Engineering and Biotechnology, New Delhi, India. Guinea pigs were infected with single-cell suspension of growing culture of either parental or Δ*citE-DM* strain via aerosol route. The extent of disease progression in guinea pigs was determined by measuring bacterial loads in lungs, spleens and histopathology analysis in lung sections as previously described (Singh et al., 2013). The extent of tissue damage in hematoxylin and eosin stained sections was quantified by determining total granuloma score and cellular infiltration as previously described (Singh et al., 2016).

### Statistical analysis

Statistical analyses were performed using Graph Pad Prism 5 software (version 5.01, GraphPad Software Inc., CA, USA). The differences between indicated groups were considered significant with *p* < 0.05.

## Author contributions

RS conceived the idea and supervised experiments. GA, DC, and SK performed *M. tuberculosis* experiments. DS performed bioinformatic analysis. RS and GA wrote manuscript with inputs from other authors.

### Conflict of interest statement

The authors declare that the research was conducted in the absence of any commercial or financial relationships that could be construed as a potential conflict of interest.

## Supplementary material

Supplementary Figure 1 Alignment of the modeled structure of CitE2 (blue) with experimentally determined structures of 3QLL (a putative citrate lyase from *Yersinia pestis*, pink) and 4ROQ (Malyl-CoA lyase from *Methylobacterium extorquens*, green).

Supplementary Figure 2 Multiple sequence alignment among CitE1, CitE2 protein and CLYBL from *Mus musculus* mitochondria was performed using Clustal Omega and edited using GeneDoc software. The highly conserved active site residues among these proteins are highlighted. Shading denotes the level of conservation among the residues.

Supplementary Figure 3 **(A)** Schematic representation of the *citE1* and *citE2* genetic loci in the wild type and *citE* mutant strains of *M. tuberculosis*. The genes *Rv2497c* and *Rv3074* are genes upstream of *citE1* and *citE2*, respectively and *Rv2499* and *Rv3076* are genes downstream of *citE1* and *citE2*, respectively. The *M. tuberculosis* strains devoid of either CitE1 or CitE2 or both homologs were constructed using the temperature-sensitive mycobacteriophages as described in Materials and Methods. **(B,C)** Characterization of mutant strains of *M. tuberculosis*. **(B)** PCR-based confirmation. The replacement of *citE1* or *citE2* with antibiotic resistance gene in *M. tuberculosis* mutant strains was confirmed by PCR using locus specific primers. **(C)** Southern blot analysis. Genomic DNAs of the wild-type and the mutant strains were digested with *Pvu* II for *citE1* and *Msc* I for *citE2*, separated on a 1.2% agarose gel, transferred to a nylon membrane, and probed with DIG-labeled locus specific probe. **(D)** Effect of deletion of *citE1* and *citE2* on transcription of their neighboring genes. qRT-PCR of *citE1* neighboring genes (Rv2497c and Rv2499c) and *citE2* neighboring genes (Rv3074 and Rv3076) was performed using mRNA isolated from mid-log phase cultures of wild type and Δ*citE*-*DM* strain. The data shown in this panel is fold change in expression of transcript levels relative to that in wild type strain obtained from two independent experiments.

Supplementary Figure 4 **(A)** Growth kinetics of various strains in cholesterol containing liquid medium. The bacterial loads of *M. tuberculosis* wild type, Δ*citE1* and Δ*citE2* were determined at time zero and day 7 of growth in Sauton's medium containing 0.01% cholesterol. **(B)** Susceptibility of wild type and single mutant strains to oxidative stress condition. Early-log phase cultures of wild type, Δ*citE1* and Δ*citE2* strains were exposed to 5 mM H_2_O_2_ for 1 day. **(C)** Influence of deletion of the *citE1* and *citE2* genes on the growth and survival of *M. tuberculosis* in macrophages. THP-1 monocytes were differentiated and infected with either wild type or Δ*citE1* or Δ*citE2*. The number of intracellular viable bacteria was determined at day 0, 4, and 7 post-infection. At designated time points 10.0-fold serial dilutions were prepared, 100 μl was plated on MB7H11 plates and incubated at 37°C for 3–4 weeks. The data depicted are mean ± S.E. obtained from at least two independent experiments.

Supplementary Figure 5 Lipid profiles of various strains of *M. tuberculosis*. **(A)** Apolar lipids were isolated from wild type and double mutant strain and resolved on TLC in petroleum ether: diethyl ether (90:10 v/v). **(B)** Apolar lipid extracts were separated by two-dimensional TLC in petroleum ether: ethyl acetate (98:2) in first direction and petroleum ether: acetone (98:2) for 2nd direction. **(C)** Apolar lipids were resolved on two dimensional TLC using petroleum ether: acetone (98:2) for 1st direction and toluene: acetate (95:5) for 2nd direction **(D**) Polar lipids isolated from various strains were resolved on two dimensional TLC's with chloroform: methanol: water (100: 14: 0.8) for 1st direction and chloroform: acetone: methanol: water (50: 60: 2.5: 3) for 2nd direction. **(E)** Polar lipids were resolved using chloroform: methanol: water (60: 30: 6) for 1st direction and chloroform: acetic acid: methanol: water (40: 25: 3: 6) for 2nd direction. For lipid detection, TLC plates were stained with either 5% phosphomolybdic acid (for apolar lipids) or 5% α-naphthol (for polar lipids). PDIM, phthiocerol dimycocerosate; TAG, triacylglycerol; MK, menaquinone; DAG, diacylglycerol; DAT, diacyltrehalose; FMA, Free mycolic acids; PIM, phosphatidylinositol mannoside (integers denote number of mannoside or acyl groups).

Supplementary Table 1 List of oligonucleotides used in the study.
