# Corrigendum: CitE Enzymes Are Essential for *Mycobacterium tuberculosis* to Establish Infection in Macrophages and Guinea Pigs

**DOI:** 10.3389/fcimb.2020.587907

**Published:** 2020-10-30

**Authors:** Garima Arora, Deepika Chaudhary, Saqib Kidwai, Deepak Sharma, Ramandeep Singh

**Affiliations:** ^1^Tuberculosis Research Laboratory, Vaccine and Infectious Disease Research Centre, Translational Health Science and Technology Institute, Faridabad, India; ^2^Symbiosis School of Biological Sciences, Symbiosis International University, Lavale, India; ^3^Manipal Academy of Higher Education, Manipal, India; ^4^Department of Biotechnology, Indian Institute of Technology Roorkee, Roorkee, India

**Keywords:** *Mycobacterium tuberculosis*, reverse TCA, β-subunit of citrate lyase, virulence, oxidative stress

Due to an oversight by the authors, there was a mistake in the Figure 7 as published. The wild type H&E stained section at 8 weeks post-infection (56 days) were replaced with the sections obtained from another animal in the same experiment. The correct Figure 7 appears below.

**Figure 7:**
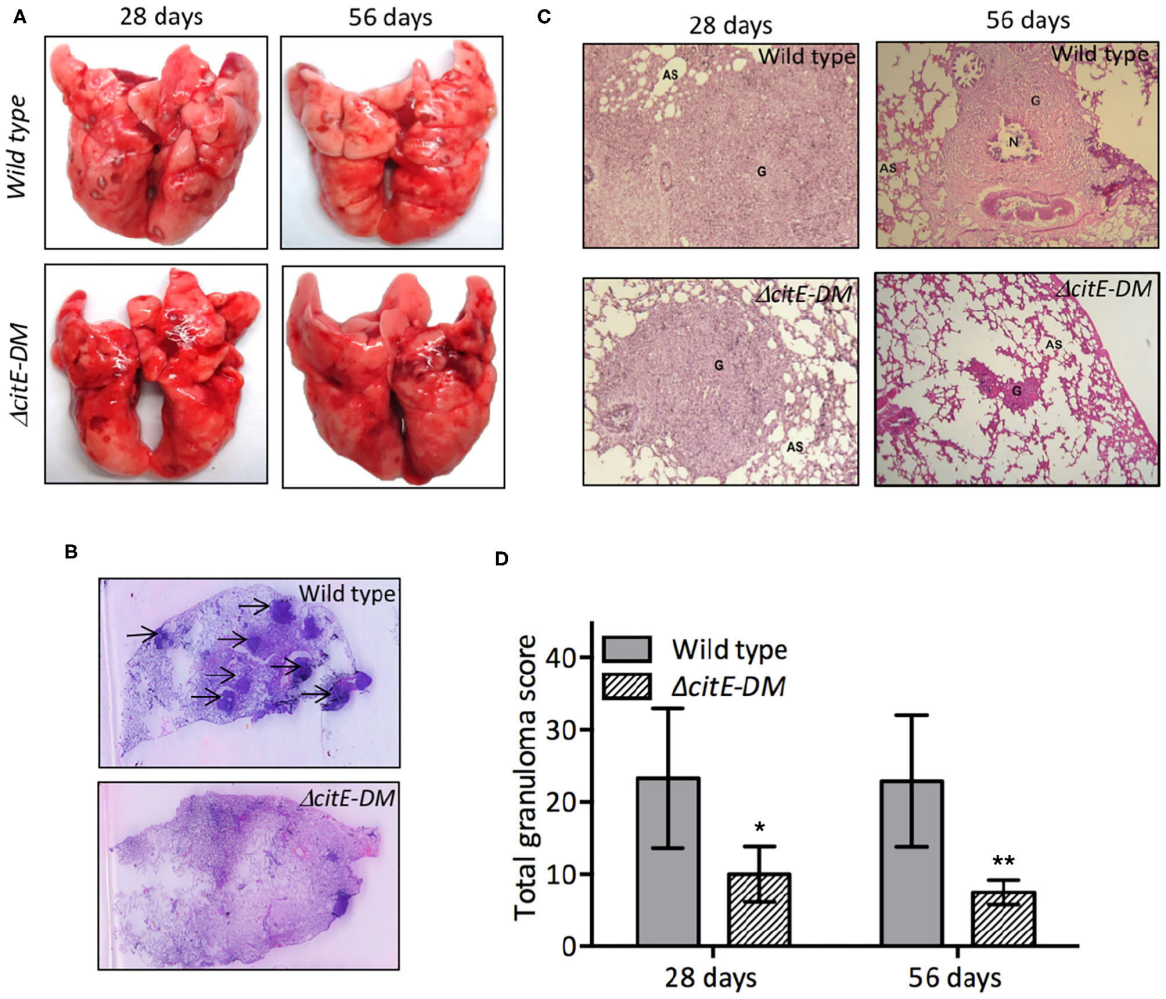
Gross pathological and histopathological analysis of lungs of infected guinea pigs. **(A)** This panel depicts representative photographs of lungs showing granulomatous lesions from guinea pigs infected with various strains at 4 or 8 weeks post-infection. **(B)** High-resolution scans (2,400 dpi) of lung sections from infected guinea pigs were performed at 8 weeks post-infection. A representative high-resolution photomicrograph for each group is shown and granulomas are marked by arrows. **(C)** Images of H & E stained lung sections from guinea pigs at day 28 and 56 post-infection. These images were taken at 40× magnification and show granulomas (G), areas of necrosis (N) and alveolar spaces (AS). **(D)** Total granuloma score in H&E-stained lung sections of animals infected with wild type or Δ*citE-DM* at both 4 and 8 weeks post-infection. Significant differences were observed for the indicated groups (paired two-tailed *t*-test, ^*^p < 0.05, ^**^p < 0.01).

The authors apologize for this error and state that this does not change the scientific conclusions of the article in any way. The original article has been updated.

