# Supplementary material for: CitE Enzymes Are Essential for Mycobacterium tuberculosis to Establish Infection in Macrophages and Guinea Pigs
Source: Front Cell Infect Microbiol. 2018 Nov 6;8:385. doi: 10.3389/fcimb.2018.00385 (PMC6232273; doi:10.3389/fcimb.2018.00385)
Supplement: Supplementary Figure 1 — Alignment of the modeled structure of CitE2 (blue) with experimentally determined structures of 3QLL (a putative citrate lyase from Yersinia pestis, pink) and 4ROQ (Malyl-CoA lyase from Methylobacterium extorquens, green). [file Presentation_1.PPTX]

## Slide 1
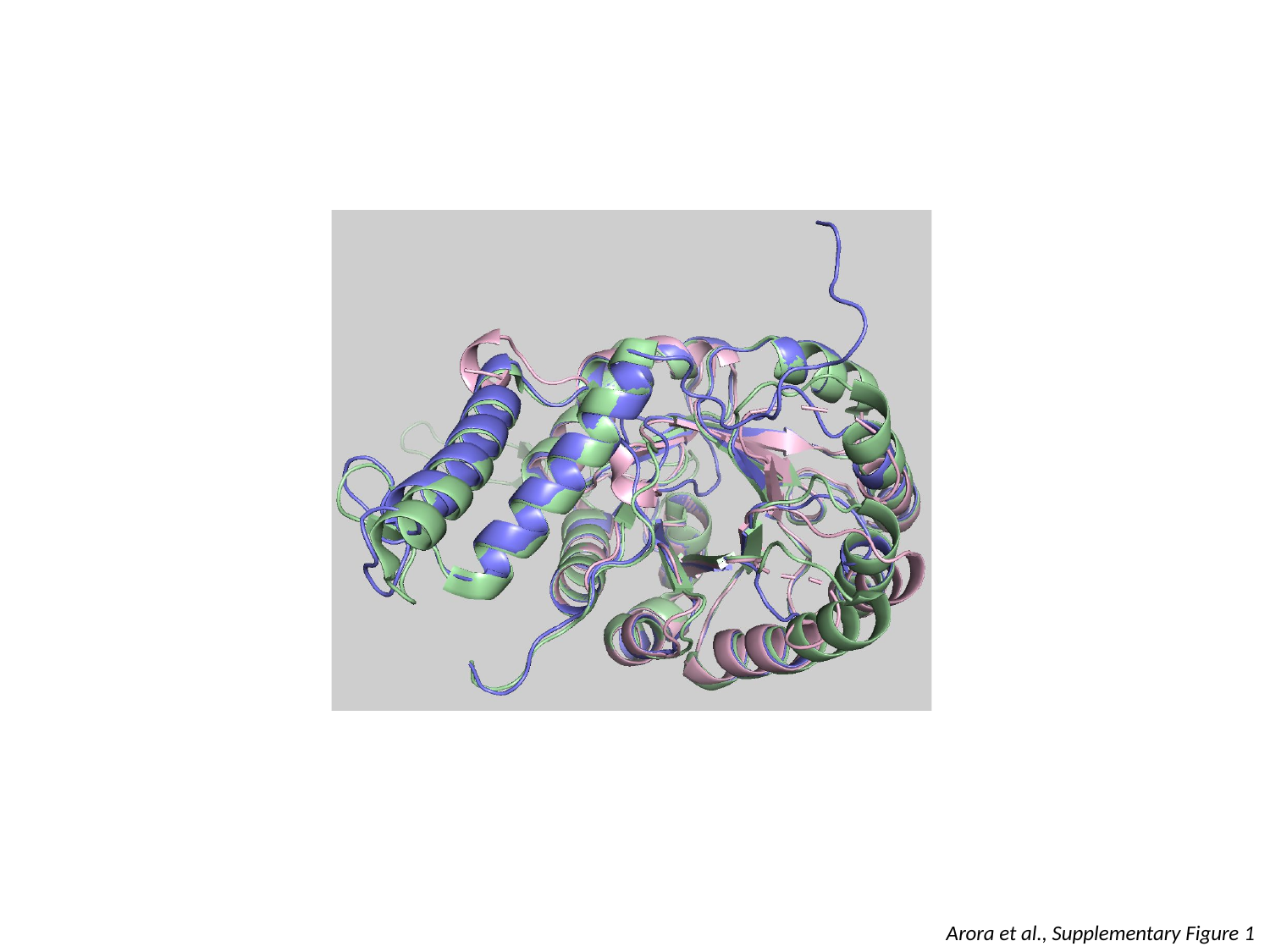

Arora et al., Supplementary Figure 1

## Slide 2
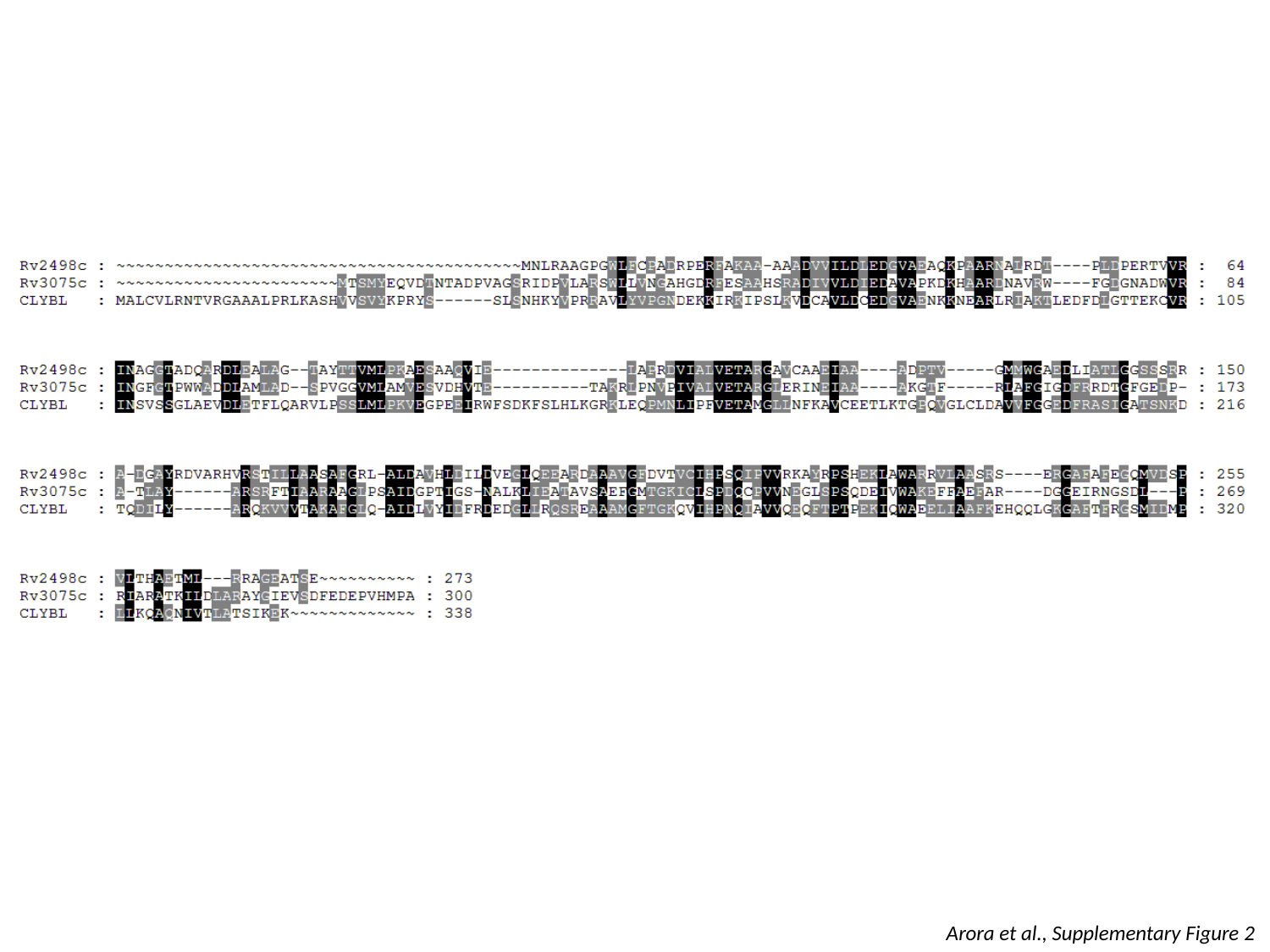

Arora et al., Supplementary Figure 2

## Slide 3
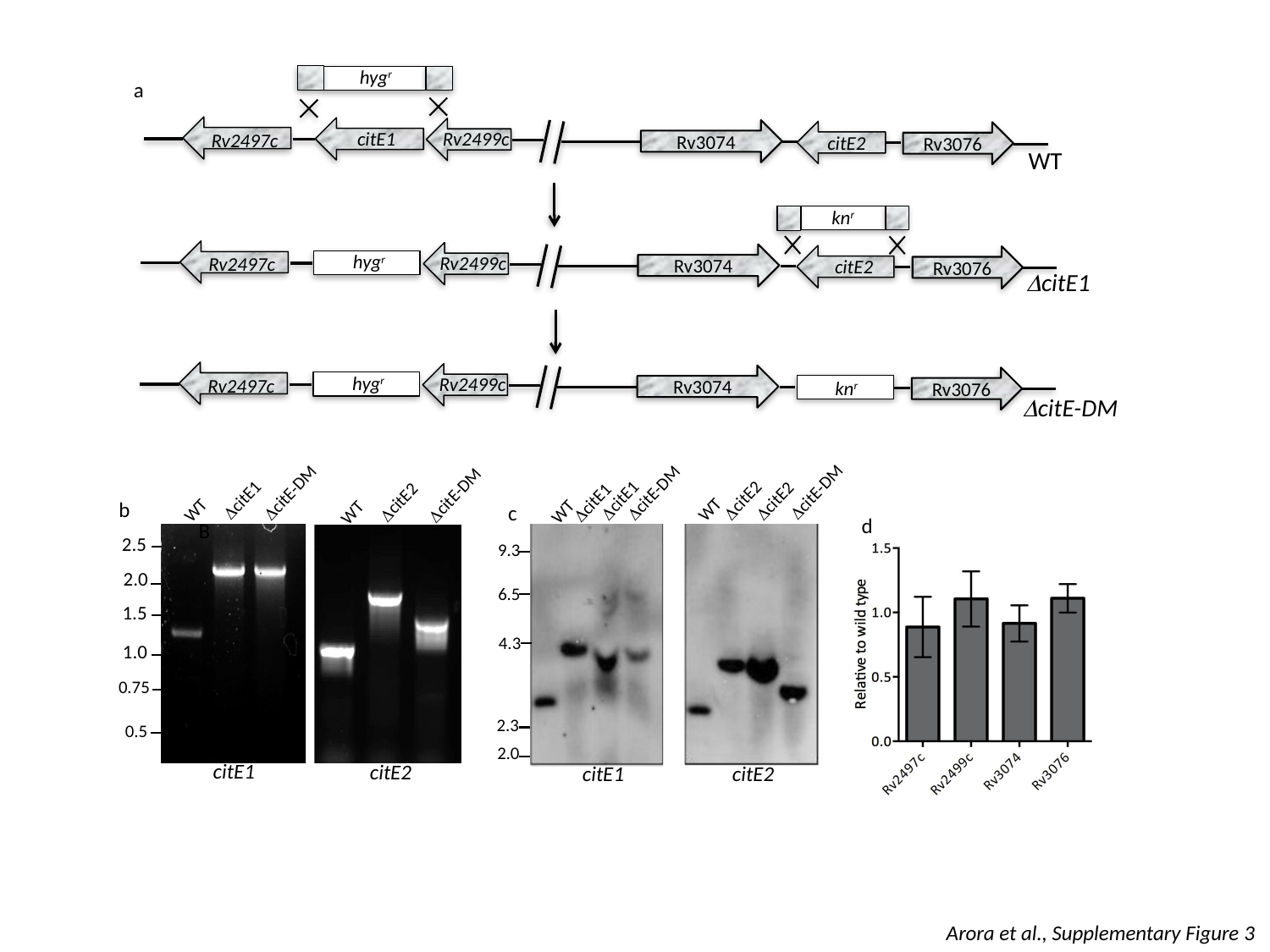

hygr
citE1
Rv3074
Rv2499c
citE1
Rv2497c
Rv3076
citE2
WT
knr
Rv3074
Rv2499c
Rv2497c
Rv3076
citE2
hygr
DcitE1
Rv3074
Rv2499c
Rv2497c
Rv3076
hygr
knr
DcitE-DM
a
DcitE-DM
DcitE2
WT
DcitE-DM
DcitE-DM
DcitE-DM
DcitE1
DcitE1
DcitE2
DcitE2
DcitE1
b
WT
WT
c
WT
d
B
9.3
6.5
4.3
2.3
2.0
2.5
2.0
1.5
1.0
0.75
0.5
citE1
citE2
citE1
citE2
Arora et al., Supplementary Figure 3

## Slide 4
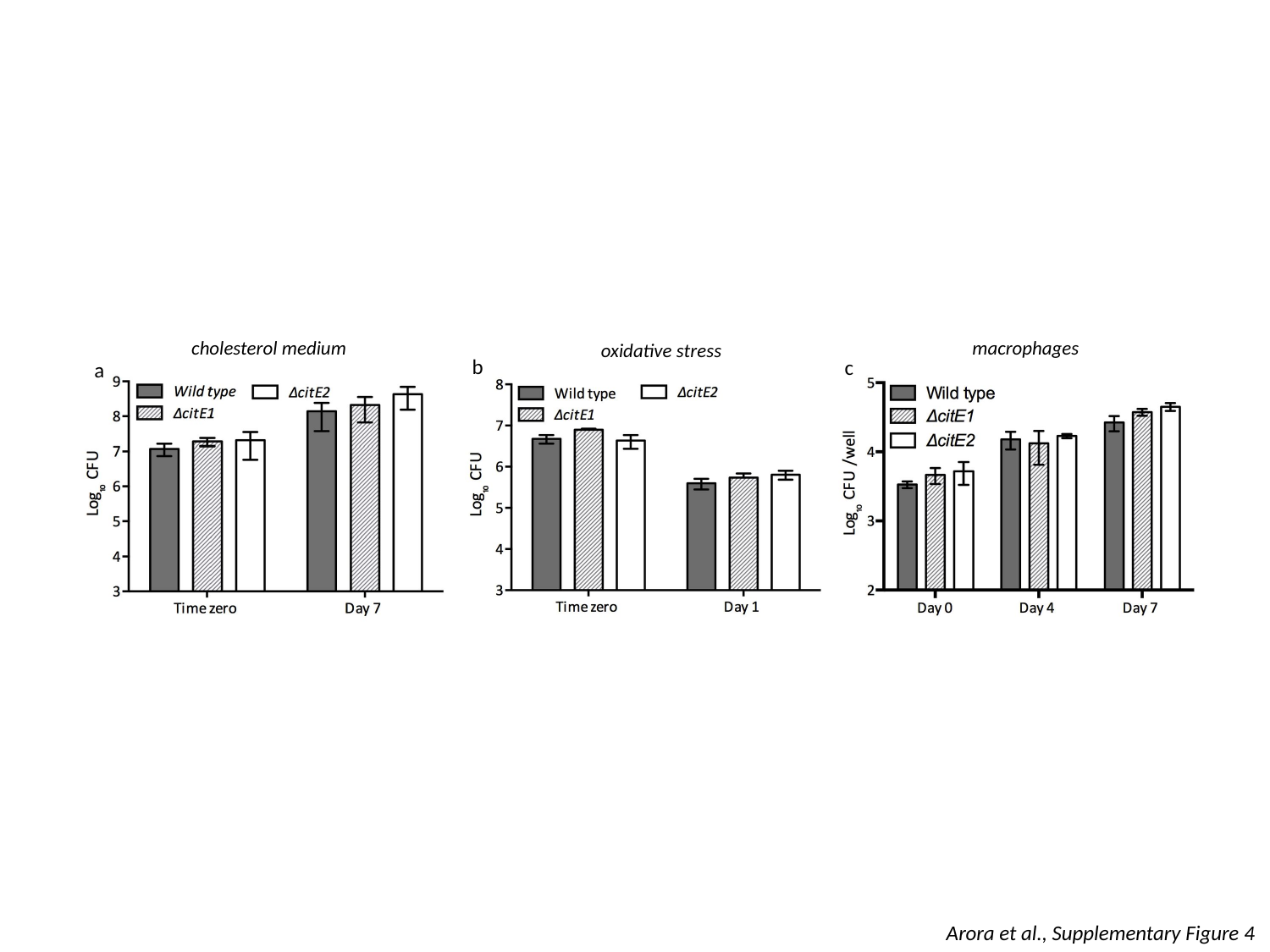

cholesterol medium
macrophages
oxidative stress
b
c
a
Arora et al., Supplementary Figure 4

## Slide 5
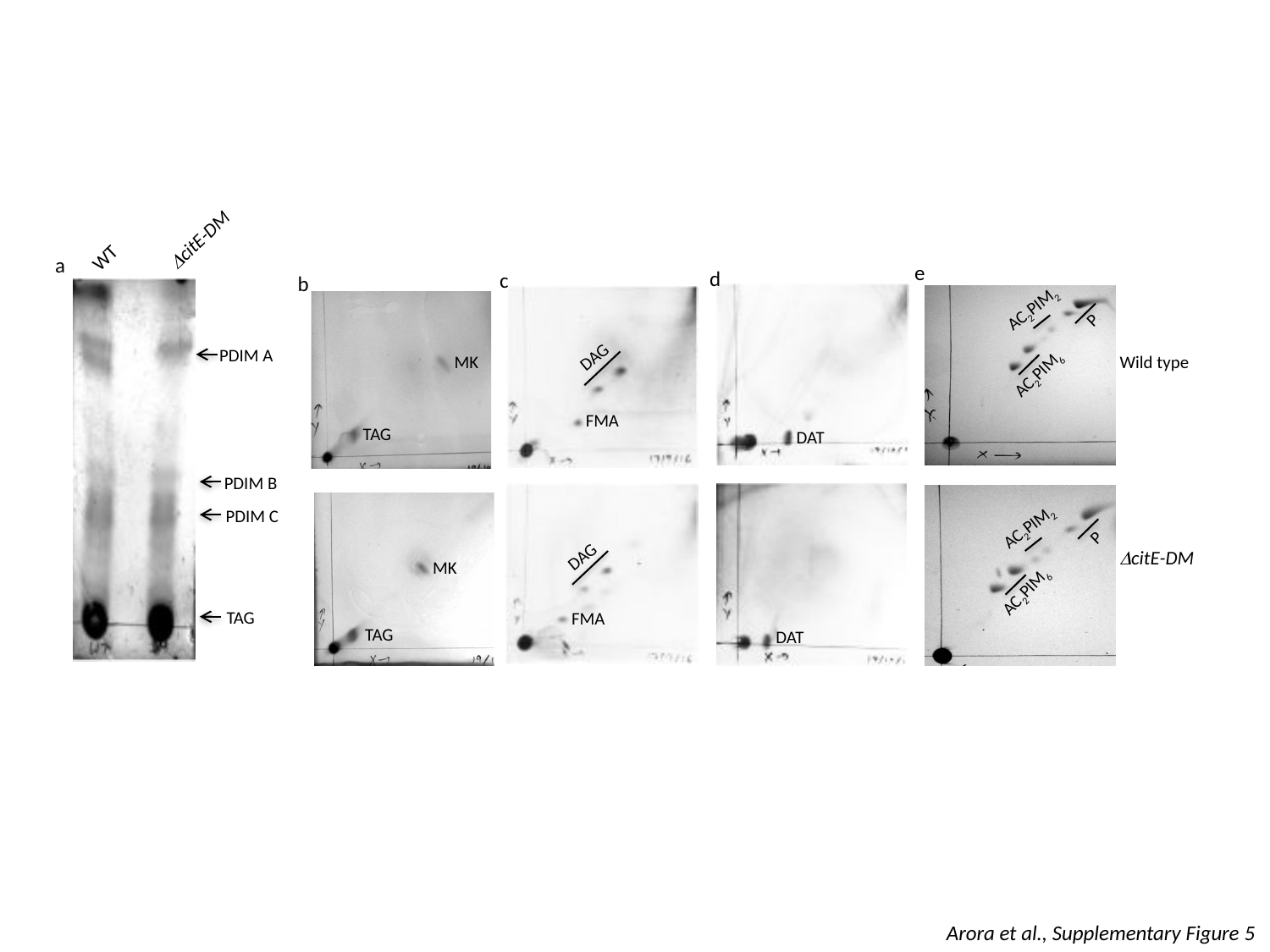

DcitE-DM
WT
a
e
d
c
b
DAG
FMA
DAT
DAG
FMA
DAT
AC2PIM2
P
PDIM A
PDIM B
PDIM C
TAG
MK
AC2PIM6
TAG
AC2PIM2
P
MK
AC2PIM6
TAG
Wild type
DcitE-DM
Arora et al., Supplementary Figure 5
