# Supplementary material for: CitE Enzymes Are Essential for Mycobacterium tuberculosis to Establish Infection in Macrophages and Guinea Pigs
Source: Front Cell Infect Microbiol. 2018 Nov 6;8:385. doi: 10.3389/fcimb.2018.00385 (PMC6232273; doi:10.3389/fcimb.2018.00385)
Supplement: Supplementary Table 1 — List of oligonucleotides used in the study. [file Table_1.docx]

**Supplementary Table 1:** List of oligonucleotides used in the study

| **Primer name** | **Forward primer (5'→3')** | **Reverse primer (5'→3')** |
| --- | --- | --- |
| *citE1* ORF | GGGCATATGCATATGATGAACCTGCGTGCCGCCGGTCCGG | GGGAAGCTTTCATTCGGAGGTGGCTTCCCCGGC |
| *citE2 ORF* | GGGCATATGCATATGATGACCTCAATGTATGAACAGGTCG | GGGAAGCTTCTAGTAGTGGTAGGTGTCAGTCGG |
| *citE1* upstream | GGGAGGCCTAGATGTTGGCGGGCAAGCCCTTC | GGGTCTAGACCACCCCGGACCGGCGGCACGCAG |
| *citE1* downstream | GGGCCATGGACCTCCGAATGAGGGCGCAGATCC | GGGACTAGTGGTGGCGCCGTCGCCCAGAAAGG |
| *citE2* upstream | gggaggcctgtctgggtgacgccgtgcagatcc | gggtctagactagtgtctcttcgctcagtcgATG |
| *citE2* downstream | gggaagcttgcggcgtatcgagcgagcgcgatgc | gggactagtctggacccgcaggccgtcgtcgacc |
| *citE1* SYBR | TGCTGATCAGGCCCGCGATCTGGAG | CGGCACCCCACATCATTCCCACGG |
| *citE2 SYBR* | atgacctcaatgtatgaacag | ccaactccgcgcgagaaccgg |
| *Rv2497c SYBR* | CGAGAATTGGGCGTCTACCTAGTGC | GCTGGTGGCGCCGTCGCCCAGAAAG |
| *Rv2499c SYBR* | AGAAGGATTCTTCAACGAGGCTTG | ACCGACCATCGTCGAGAGAGTGAAC |
| *Rv3074 SYBR* | GCCACACACGCTGGCCGCCCTG | GCGGTCAGATACGTCATGGTGTC |
| *Rv3076 SYBR* | GCGGTCAGATACGTCATGGTGTC | GACCGCTTGGCCATGGCGCGGCC |
| *sigA SYBR* | acgaagaccacgaagacctcgaa | gtaggcgcgaaccgagtcggcgg |
